# Indeterminate *tcdB* using a *Clostridium difficile* PCR assay: a retrospective cohort study

**DOI:** 10.1186/1471-2334-13-324

**Published:** 2013-07-16

**Authors:** Jerome A Leis, Wayne L Gold, John Ng, Zahir Hirji, Dylan R Pillai, George Broukhanski, Paula Raggiunti, Susy Hota, Allison McGeer, Susan M Poutanen

**Affiliations:** 1 Division of Infectious Diseases, Department of Medicine, University Health Network/Mount Sinai Hospital, Toronto, ON, Canada; 2 Division of Infectious Diseases, Department of Medicine, University of Toronto, Toronto, ON, Canada; 3 Department of Microbiology, University Health Network/Mount Sinai Hospital, Toronto, ON, Canada; 4 Bridgepoint Hospital, Toronto, ON, Canada; 5 Division of Medical Microbiology, Department of Laboratory Medicine and Pathobiology, University of Toronto, Toronto, ON, Canada; 6 Public Health Ontario, Toronto, ON, Canada; 7 Rouge Valley Health System, Toronto, ON, Canada; 8 Current address: Calgary Lab Services and Department of Pathology and Laboratory and Medicine, University of Calgary, Calgary, AB, Canada

**Keywords:** *Clostridium difficile*, Polymerase chain reaction, Toxin B gene, False negative results

## Abstract

**Background:**

*C*. *difficile* (CD) real-time polymerase chain reaction (PCR) for toxin B gene (*tcdB*) is more sensitive, and reduces turnaround time when compared to toxin immunoassay. We noted typical amplification curves with high *tcdB* cycle thresholds (Ct) and low endpoints (Ept) that are labeled negative by the Xpert® *C*. *difficile* assay (Cepheid) and undertook this study to determine their significance.

**Methods:**

We defined an indeterminate CD assay result as detection of a typical PCR amplification curve with an Ept >10 that was interpreted as negative by the Xpert® assay. Samples with indeterminate Xpert® result were collected for 5 months and retested by Xpert®, cultured for toxigenic CD, and isolates subjected to PCR ribotyping, detection of toxin genes and multilocus variable-number tandem repeat analysis (MLVA) typing. Chart reviews were completed to assess if patients met the Society of Healthcare Epidemiology of America and the Infectious Diseases Society of America CD infection (CDI) clinical case definition. Illness severity was compared with *tcdB* Ct and culture results.

**Results:**

During the 5-month study period, 48/3620 (1%) of specimens were indeterminate and 387/3620 (11%) were positive. Of the 48 patients with indeterminate results, 39 (81%) met the clinical case definition of CDI, and 7 of these (18%) met criteria for severe CDI. Toxigenic stool cultures were positive for 86% (6/7) of patients with severe CDI, 19% (6/32) of patients with non-severe CDI, and 44% (4/9) of patients who did not meet the clinical case definition of CDI (p = 0.002). Lower *tcdB* Ct and higher Ept were associated with greater likelihood of toxigenic culture positivity (p = 0.03) and more severe symptoms (p = 0.06). Indeterminate results were not associated with a particular technologist or instrument module, or CD strain type.

**Conclusions:**

A subset of specimens (1%) using the Xpert® *C*. *difficile* assay have typical amplification curves and are interpreted as negative. At least one-third of these results are associated with positive CD culture. The mechanism of these indeterminate results is not technique-related, equipment-related, or due to particular CD strains. Clinicians should be aware that even PCR testing has the potential to miss CDI cases and further highlights the importance of clinical context when interpreting results.

## Background

*Clostridium difficile* is a significant nosocomial and community-acquired pathogen associated with morbidity, mortality and cost to the health-care system [1]. Diagnosis of *Clostridium difficile* infection (CDI) relies on clinical manifestations of diarrhea, fever, abdominal pain and leukocytosis, supported by laboratory confirmation of toxigenic *C*. *difficile* in stool. *C*. *difficile* toxin real-time polymerase chain reaction (PCR) is increasingly being adopted in clinical laboratories because it is more sensitive and reduces turnaround time when compared to toxin immunoassay [2]. The Xpert® *C*. *difficile* assay (Cepheid, Sunnyvale, CA) is a real-time random-access PCR assay which detects the toxin B gene (*tcdB*). It has a sensitivity of 93.5-100% and specificity of 94.0-97.9% compared with toxigenic culture [2-5].

According to the product monograph, the amplification curve of the Xpert® *C*. *difficile* assay must have a cycle threshold within a valid range and endpoint above a set minimum to be considered positive for *tcdB*. During our evaluation of this assay, we noticed typical amplification curves for some specimens that had high cycle thresholds and low endpoints, which were interpreted as negative by the Xpert® assay. We undertook the following quality assurance study to investigate the clinical and microbiologic significance of these results.

## Methods

### Setting and study design

In January 2011, real-time PCR assay for detection of *C*. *difficile* toxin B (Cepheid GeneXpert System, Sunnyvale, CA) was introduced at the University Health Network/Mount Sinai Hospital Department of Microbiology clinical laboratory in Toronto, Canada. This laboratory services six acute care hospitals, three inpatient rehabilitation centres and one hospital providing complex care.

### Definitions

We defined an indeterminate Xpert® *C*. *difficile* assay result as detection of a typical PCR amplification curve with an endpoint >10 that was interpreted as negative by Xpert®. Between January 5 and May 18, 2011, all non-duplicate Xpert® *C*. *difficile* assay results that displayed an indeterminate result were included in the study.

### Clinical data collection

Infection prevention and control records and patient medical records were reviewed to determine whether patients with indeterminate results met the clinical case definition of CDI as defined by the Society of Healthcare Epidemiology of America and the Infectious Diseases Society of America (SHEA/IDSA) [6]. To meet this case definition, patients required passage of three or more unformed stools in less than 24 hours that was not due to an alternate cause. Patients were classified as having severe disease if they had a score of ≥2 points where 1 point each was given for age >60 years, temperature >38.3°C, serum albumin <25 g/L, or peripheral white blood cell count >15.0 × 10^9^/L, and 2 points were given for endoscopic evidence of pseudomembranous colitis or requirement for intensive care unit admission [7]. If same-day blood results were not available, results from blood samples obtained within 48 hours of the stool specimen were accepted. Outcomes assessed included the need for intensive care unit admission or colectomy due to the CDI episode and patient status at 30 days (deceased, alive in hospital or discharged from hospital).

### Microbiologic studies

Laboratory records were reviewed to identify whether indeterminate results were associated with a particular technologist or a particular instrument module. Each stool specimen with an indeterminate result was planted onto *C*. *difficile* moxalactam-norfloxacin agar, with toxigenicity of isolated *C*. *difficile* confirmed by a laboratory-derived *tcdB* PCR assay [8,9]. Each indeterminate specimen was subjected to repeat PCR testing using Xpert® on an aliquot of frozen (−80°C) stool to assess whether repeat testing of indeterminate results could improve detection of *C*. *difficile* compared with toxigenic culture.

To determine whether indeterminate results were associated with a particular *C*. *difficile* strain, toxigenic culture isolates were subjected to PCR ribotyping, toxin genotyping and multilocus variable-number tandem repeat analysis (MLVA) typing as previously described [8,9]. Ribotypes were identified using http://webribo.ages.at/[10] and data analysed using BioNumerics software v.6.6 (Applied Maths, Austin, TX, U.S.A).

### Statistical methods

Data were analyzed using Microsoft Excel, version 11.0, to provide descriptive statistics related to the incidence of indeterminate *C*. *difficile* results and the presence of CDI based on our preselected clinical criteria. Statistical analysis was performed using Chi-squared or Fisher’s exact tests using GraphPad software Inc., to assess the statistical significance of differences in proportions.

### Ethics

Approval from the local research ethics board was waived because the clinical isolates were obtained as part of standard care and the project was conducted for microbiology quality assurance purposes.

## Results

During the 5-month study period, 48 of 3620 (1%) of specimens were indeterminate and 387 of 3620 (11%) were positive. All indeterminate results were associated with inpatient admissions or emergency visits and occurred throughout the ten health care facilities serviced by the laboratory. They were not associated with one technologist or one instrument module.

### Clinical features

Thirty-nine of 48 patients (81%) with indeterminate results met the clinical criteria for CDI while 9 (19%) patients had less than three bowel movements within 24 hours or had an obvious alternate cause for diarrhea such as a flare of inflammatory bowel disease, another bacterial cause of diarrhea or laxative use.

Of those who met the clinical case definition of CDI, the median age was 72 years (18–93 years), 21 (54%) were female, 4 (10%) had fever (T ≥ 38.3°C), 4 (10%) had leukocytosis (WBC >15.0 × 10^9^/L), 8 (21%) had hypoalbuminemia (< 25 g/L), and 5 (13%) had acute kidney injury (serum creatinine >1.5 × baseline). Seven (18%) patients met our criteria for severe disease; none suffered a CDI-related complication. Three patients (all with non-severe disease) underwent endoscopy; none had evidence of pseudomembranous colitis. Six patients who met CDI clinical criteria had a documented positive *tcdB* result using Xpert® within 30-days of the indeterminate result; three of these occurred before and three after the indeterminate result.

In terms of therapy, 24/39 (62%) received oral metronidazole, 3/39 (8%) received oral vancomycin, 6 (15%) received both oral vancomycin plus oral or intravenous metronidazole, and 6 (15%) received no therapy. There was no difference between patients in each treatment group in terms of disease severity or toxigenic culture-positivity.

### Microbiologic studies

Sixteen of the 48 samples (33%) with indeterminate results yielded toxigenic *C*. *difficile* in culture. Stool specimens from 6 of the 7 patients (86%) with severe CDI were positive by toxigenic stool culture compared with 6 of 32 patients (19%) with CDI who did not meet criteria for severe disease and 4 of 9 patients (44%) who did not have CDI by clinical criteria (P = 0.002).

**Figure 1:**
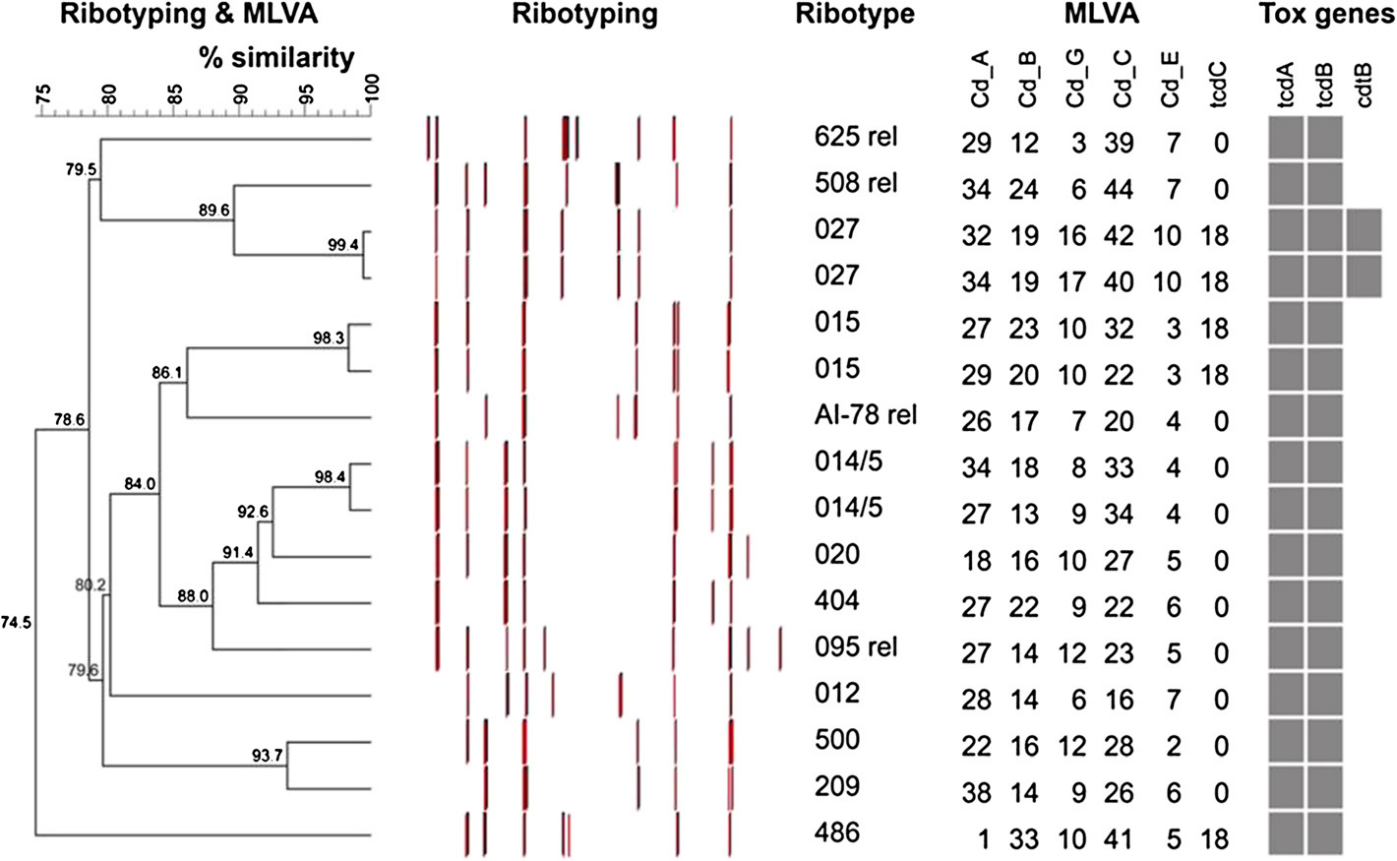
**Ribotyping and multiple loci variable-number tandem-repeat analysis (MLVA) results for *C. difficile *isolated from stool specimens with indeterminate Xpert**® ***C. difficile *assay results.** % similarity was calculated using ribotyping and MLVA composite data sets using the unweighted pair group method with arithmetic mean. rel = related.

As demonstrated in Table 1, lower cycle thresholds were associated with a greater likelihood of toxigenic culture positivity (P = 0.03) and more severe clinical disease (P = 0.06). Repeat testing of indeterminate specimens resulted in conversion to negative in 51% of cases and positive in 20% of cases (Table 2). Culture-positive specimens were more likely to be positive on repeat testing compared to culture-negative specimens (P = 0.03). Results of ribotyping and MLVA revealed that indeterminate results were not associated with a particular *C*. *difficile* strain (Figure 1).

**Table 1:** **Proportions (%) of stool specimens with indeterminate Xpert**® ***C. difficile *assay results that yielded toxin-producing *C. difficile *in culture stratified by *tcdB *cycle-threshold and clinical severity**

**Xpert® *C. difficile *assay *tcdB *cycle-threshold**	**Patients with indeterminate results (n = 48)**	**Patients who did not meet the *C. difficile *infection clinical case definition (n = 9)**	**Patients who met the *C. difficile *infection clinical case definition with non-severe clinical disease (n = 32)**	**Patients who met the *C. difficile *infection clinical case definition with severe clinical disease (n = 7)**
37.1-37.9	6/11 (55)	0/2 (0)	1/4 (25)	5/5 (100)
38.0-38.9	6/16 (38)	1/3 (33)	4/11 (36)	1/2 (50)
39.0-30.9	3/16 (19)	2/3 (67)	1/13 (8)	0
≥40	1/5 (20)	1/1 (100)	0/4 (0)	0

**Table 2:** **Results of repeat Xpert**® ***C. difficile *testing of indeterminate specimens for all specimens, culture-positive specimens, and specimens from patients with severe disease**

**Result**	**Original clinical laboratory result (N (%))**	**Repeat test (N (%))†**	**Repeat test of culture-positive specimens (N (%))**	**Repeat test of specimens from patients with severe CDI (N (%))**
Negative	0 (0)	23/45 (51)	2/14 (14)	2/7 (29)
Indeterminate*	48 (100)	13/45 (29)	7/14 (50)	2/7 (29)
Positive	0 (0)	9/45 (20)	5/14 (36)	3/7 (43)

* These results were interpreted as negative by the Xpert® assay but had typical amplification curves with a high cycle threshold and low endpoint.

†Three samples did not have sufficient quantity of stool to repeat testing.

## Discussion

The Xpert® *C*. *difficile* assay is a highly sensitive real-time random-access PCR assay. We describe a subset of clinical stool specimens (~1%) with indeterminate results for *tcdB* which may be clinically significant. Indeed, one-third had toxigenic *C*. *difficile* culture confirmation and over 80% met the SHEA/IDSA clinical case definition of *C*. *difficile* infection, 18% of whom had features of severe infection. We cannot rule out that some of these patients may have been colonized with low levels of *C*. *difficile* while having another cause for their diarrhea other than *C*. *difficile* that was not identified by their care providers. Previous published work has emphasized the high sensitivity of PCR for *C*. *difficile* toxin B detection and the potential to over-diagnose CDI when interpreting *C*. *difficile* PCR results without considering clinical data [11-14]. This study emphasizes that clinicians should be aware that even PCR testing has the potential to miss CDI cases on rare occasions and further highlights the importance of clinical context when interpreting test results.

The observation that toxigenic *C*. *difficile* may be present at or below the limit of detection of the Xpert® *C*. *difficile* assay has recently been noted in a study evaluating its use for detection of environmental contamination [15]. In this study, only one-third of environmental specimens positive by toxigenic *C*. *difficile* culture were detectable as positive by the Xpert® *C*. *difficile* assay. Our results suggest that low levels of *C*. *difficile* may also be present *in vivo* that fall below the level of detection of PCR.

The mechanism of these indeterminate results is not technique-related, equipment-related, or due to particular *C*. *difficile* strains. Other potential factors that could contribute to indeterminate results, but were not specifically evaluated by this study, include the timing of therapy for *C*. *difficile* in relation to testing and the presence of PCR interfering substances in stool. Sunkesula et al. recently showed that in a cohort of 51 PCR confirmed CDI patients on treatment, 14% of patients had converted to negative PCR within 24 hours, which increased to 35% of patients at 48 hours [16]. Any significant delay in obtaining a patient specimen following initiation of therapy could contribute to a false-negative *tcdB* by PCR [16,17].

It is worth considering whether indeterminate results should be routinely reported by the microbiology laboratory, or whether this information could be used to improve performance characteristics of the Xpert® *C*. *difficile* assay. Changing the instrument’s threshold for reporting positives could increase sensitivity but at the cost of specificity. Repeat testing of indeterminate specimens would be associated with increased cost and may not significantly improve performance characteristics as it produced mixed results in our cohort. Although culture-positive specimens were more likely to be positive on repeat testing, two patients who converted to negative were culture-positive and had severe disease. Given these limitations, our institution currently reports all specimens that are negative by the Xpert® assay but have typical amplification curves with endpoints >10 as indeterminate, with a report that recommends interpretation based on the clinical context. These patients are assessed by infection control practitioners and contact precautions are enforced for those patients with clinically significant diarrhea.

This study has several important limitations including its small sample size and retrospective design. We applied pre-selected published criteria to determine disease severity but many features including leukocytosis and hypoalbuminemia are not specific for CDI and may be present in acutely ill patients for a variety of reasons. We did, however, use infection prevention and control records and patient medical records to determine whether patients had clinically significant diarrhea, and those with alternate causes for their symptoms based on available clinical and microbiologic data, were excluded. Another limitation is that we did not account for the timing of therapy for *C*. *difficile* in relation to testing, which may have played a role in producing indeterminate results [16,17]. Further work is needed to better understand the mechanism of indeterminate PCR results, their clinical importance and their potential role in hospital transmission.

## Conclusions

A subset of stool specimens (1%) using the Xpert® *C*. *difficile* assay have typical amplification curves and are interpreted as negative. At least one-third of these results are associated with positive CD culture. Our study, while limited by its small sample size and retrospective design, emphasizes that clinicians should be aware that even PCR testing has the potential to miss CDI cases and further highlights the importance of clinical context when interpreting results.

## Competing interests

The authors declare that they have no competing interest.

## Authors’ contributions

All authors contributed significantly to this manuscript. SH, AM, JN and SMP were involved in the conception of the project, data analysis and editing of the manuscript. JAL undertook the chart review, data analysis and drafted the manuscript. WLG was involved in the data analysis and contributed significantly to the editing of the manuscript. DP and GB performed toxigenic stool testing, PCR-ribotyping and multiple-locus variable number tandem repeat analysis, and both edited the manuscript. PR and ZH were involved in retrieving infection control records of patients and edited the manuscript. All authors approved the final version of the manuscript.

## Pre-publication history

The pre-publication history for this paper can be accessed here:

http://www.biomedcentral.com/1471-2334/13/324/prepub
